# Evaluation of hydrophilic surface osseointegration in low-density bone: Preclinical study in rabbits

**DOI:** 10.1590/0103-6440202305352

**Published:** 2023-07-17

**Authors:** Gustavo da Col Santos Pinto, Isadora Aparecida Ribeiro dos Reis, Amanda de Carvalho Silva Leocádio, Matusalem Silva, Rafael Silveira Faeda, Guilherme José Pimentel Lopes de Oliveira, Elcio Marcantonio

**Affiliations:** 1 São Paulo State University (Unesp), School of Dentistry, Araraquara, Department of Diagnosis and Surgery, Araraquara, Brazil.; 2 Department of Periodontology, Federal University of Uberlândia, Uberlândia, Brazil.; 3 Instituto Latino-americano de pesquisa odontológica (ILAPEO), Curitiba, Brazil.; 4 University of Araraquara (Uniara), School of Dentistry, Post-graduation im Implantology, Araraquara, Brazil.

**Keywords:** bone-implant interface, dental implants, osseointegration

## Abstract

The aim of this study was to evaluate the osseointegration of a hydrophilic surface (blasting + acid etching + immersion in isotonic solution) in comparison with that of a control surface (blasting + acid etching) using an experimental model of low-density bone. To perform the study, 24 rabbits were submitted to the installation of 4 implants in the iliac bone bilaterally: 2 implants with a control surface and 2 implants with a hydrophilic surface. The rabbits were euthanized at 2, 4, and 8 weeks after implant installation. After euthanasia, one implant from each surface was used to perform the removal torque analysis, and the other implant was used for the execution of non-decalcified histological sections and evaluation of the bone implant contact (% BIC) as well as the fraction of bone tissue area between the implant threads (% BBT). The implants with a hydrophilic surface presented higher %BIC (42.92 ± 2.85% vs. 29.49 ± 10.27%) and % BBT (34.32 ± 8.52% vs. 23.20 ± 6.75%) (p < 0.05) in the 2-week period. Furthermore, the hydrophilic surface presented higher removal torque in the 8-week period (76.13 ± 16.00 Ncm2 vs. 52.77 ± 13.49 Ncm2) (p<0.05). Implants with a hydrophilic surface exhibited acceleration in the process of osseointegration, culminating in greater secondary stability in low-density bone than in implants with a control surface.

## Resumo

O objetivo deste estudo foi avaliar a osseointegração de uma superfície hidrofílica (jateamento + ataque ácido + imersão em solução isotônica) em comparação com uma superfície controle (jateamento + ataque ácido) usando um modelo experimental de osso de baixa densidade. Para realizar o estudo, 24 coelhos foram submetidos a instalação de 4 implantes bilateralmente no osso ilíaco: 2 implantes com superfície controle e 2 implantes com superfície hidrofílica. Os coelhos foram eutanasiados com 2, 4 e 8 semanas após a instalação dos implantes. Após a eutanásia, um implante de cada superfície foi usado para avaliar o torque de remoção, e o outro implante foi utilizado para execução de cortes histológicos não descalcificados e avaliação de contato osso implante (% BIC) bem como a fração da área tecido ósseo entre as roscas do implante (% BBT). Os implantes com superfície hidrofílica apresentaram maior %BIC (42.92 ± 2.85% vs. 29.49 ± 10.27%) e % BBT (34.32 ± 8.52% vs. 23.20 ± 6.75%) (p < 0.05) no período de 2 semanas. Além disso, a superfície hidrofílica apresentou maior torque de remoção no período de 8 semana (76.13 ± 16.00 Ncm2 vs. 52.77 ± 13.49 Ncm2) (p<0.05). Implantes com a superfície hidrofílica apresentaram aceleração no processo de osseointregração, culminando em melhor estabilidade secundária no osso de baixa densidade em relação a implantes com superfície controle.

## Introduction

The osseointegration process is the basis for the high success rates of implant-supported rehabilitations. Therefore, prostheses supported by dental implants have been preferentially indicated for the rehabilitation of different patterns of edentulous areas ^1^. However, despite the high success rates of osseointegrated implants, some factors have been related to delays and/or failures in the osseointegration process ^2^ ^,^ ^3^. Among these factors, it has been indicated that systemic diseases (e.g., diabetes) ^5^, the use of anti-resorptive drugs (e.g., bisphosphonates) ^3^, the habit of smoking ^2^, and bone quality at the site indicated for implant placement ^5^ are related to impaired bone healing, which may interfere with early or immediate occlusal loading planning.

The shortest time required for functional rehabilitation is a goal in implant therapy, as immediate loading reduces the total rehabilitation time of these patients ^6^ ^,^ ^7^. However, in low-density bone situations, the application of immediate loading is difficult due to the insufficient primary stability obtained with implants placed in these regions, making it necessary to wait for the conversion of primary stability into secondary stability ^7^ ^,^ ^8^.

Some modifications in the dental implant surface have been shown to accelerate osseointegration ^9^, which enables faster rehabilitation even in challenging clinical conditions ^10^ ^,^ ^11^. The hydrophilic implant surface presents a high degree of wettability ^9^, increasing the proliferation of undifferentiated mesenchymal cells ^12^ which are subsequently stimulated to secrete and express osteogenic factors ^11^ ^,^ ^13^. Among these surfaces, the double blasting and acid etched surface, manufactured in an environment with the absence of atmospheric oxygen, has been highlighted (9, 13). This surface has been shown to accelerate osseointegration in native bone ^14^, and in grafted areas ^10^ in preclinical studies.

These abovementioned properties of the hydrophilic surface, in theory, may contribute to accelerating the conversion from primary to secondary stability, reducing rehabilitation time in challenging situations, such as the presence of low-density bone. Thus, the aim of this study was to evaluate the effect of a hydrophilic surface on the osseointegration process in low-density bone. The null hypothesis is that the hydrophilic surface and the control surface will demonstrate the same potential to achieve the osseointegration process.

## Material and methods

### Experimental model

This project was carried out in accordance with the Ethical Principles for Animal Experimentation, adopted by the Brazilian College of Animal Experimentation (COBEA), after approval by the Animal Ethics Committee of our institution, number 11/2016. For the present research, 24 male New Zealand Albino rabbits were used, aged approximately 5 months and weighing between 4 and 5 kilograms. The animals were provided by the Central Vivarium of our institution, in an environment with a temperature between 22 and 24ºC, and a controlled light cycle (12 hours light and 12 hours dark), and solid feed and water were provided *ad libitum* throughout the experimental period. A period of 30 days was respected for acclimatization of the animals to the vivarium.

### Experimental design

**Figure 1:**
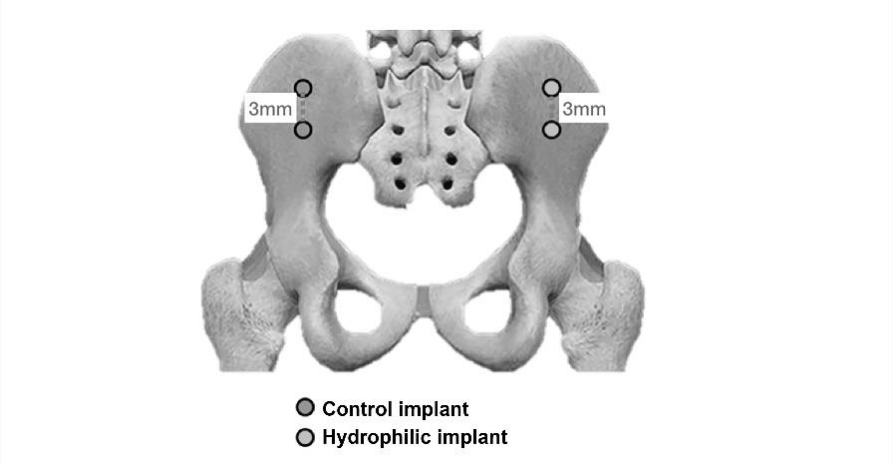
Distribution of implants with different surface treatments in iliac bone. Two implants with control surfaces were installed in the left iliac, and 2 implants with hydrophilic surfaces were installed in the right iliac.

To evaluate the influence of the different microstructures of titanium implants in the osseointegration process, the 24 rabbits were randomly divided into 3 experimental periods (2, 4, and 8 weeks). Two types of implant surfaces were evaluated: Control surface (NP - sandblasting + acid etching - NeoPoros ® Surface, Neodent, Curitiba, Brazil) and hydrophilic surface (AQ - sandblasting + acid etching + immersion in isotonic solution of 0.9% sodium chloride - Surface Acqua®, Neodent, Curitiba, Brazil). Each animal received 4 short-implants (Neodent Osseointegrable Implant, Neodent, Curitiba, PR, Brazil), 4 mm in diameter x 5 mm in height: 2 hydrophilic surface implants (AQ) were installed in the iliac bone on the right side and 2 control surface (NP) implants were placed on the left side (Figure 1).

### Surgical procedure

Initially, the animals were weighed and anesthetized intramuscularly with a combination of ketamine (Quetamina Agener®, Agener União SA - 0.35 mg / kg) and xylazine (Dopaser® Laboratorios Calier SA Barcelona, Spain - 0.5 mg / kg). Subsequently, trichotomy was performed in the right and left dorsal regions of the iliac rabbit bone, followed by antisepsis with iodine-povidone. Local anesthesia (2% mepivacaine hydrochloride + adrenaline 1: 100,000 - Scandicaíne ® 2% - Spécialités Sptodont, Sain - Maur, France) was also applied in the region to allow peripheral vasoconstriction by reducing local bleeding and optimizing the surgical procedure. Next, with a scalpel blade (nº15), a dermo-periosteal incision of approximately 5 cm in length was performed. This incision allowed the detachment and exposure of the iliac bone. The preparation for implant installation was performed on the right and left sides, and the bone was milled with metal drills under heavy physiological saline cooling. The drill sequence recommended by the manufacturer was followed for installation of implants 4.0 mm in diameter and 5.0 mm in height. The drillings started with a drill spear, followed by drills 2.0, 2/3, 2.8, 3.15, and 3.3. The implants were initially installed at low speed with counter-angle and manually terminated, with the aid of the wrench until primary stability was obtained, and then the cover screws were installed.

Two implants with control surfaces were installed in the left iliac, and 2 implants with hydrophilic surfaces were installed in the right iliac, maintaining a separation distance of 3 mm. All the regions where the implants were placed were classified as type IV bone (very thin layer of cortical bone with low density trabecular bone of poor strength). Implants installed in the anterior region of each iliac were submitted to the removal torque test, while the posterior implants of both sides were submitted to histometric tests (% BIC and % BBT) through non-decalcified histological sections

After the surgery, all animals received a single dose of antibiotic (Pentabiotico®, Wyeth-Whitehall Ltda, São Paulo, Brazil - 0.1 ml / kg) and Tramadol (dose: 5 mg / kg IM). The animals were euthanized through anesthetic overdose 2, 4, and 8 weeks postoperatively, according to the experimental periods of each group.

### Biomechanical evaluation

**Figure 2:**
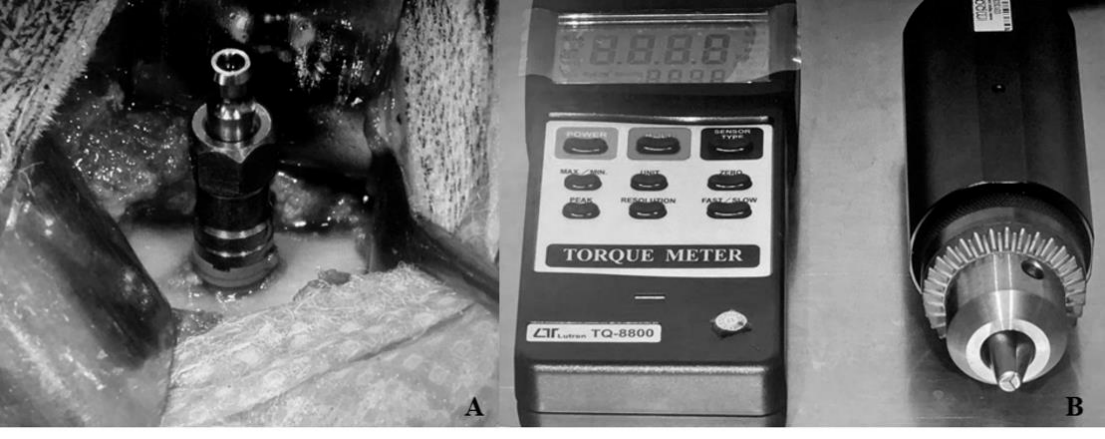
Representation of the biomechanical analysis that was assessed by the insertion and removal torque test. A) Implants placed in the native bone; B) The torque wrench used to apply the counterclockwise movement to remove the implants and obtain the removal torque forces

At the moment of implant placement, the insertion torque was measured. After euthanasia, in each period of analysis (2, 4, and 8 weeks), the implants were removed. The bone samples were stabilized, and a hexagonal wrench was connected to both the implant and the torque wrench (Lutron, model TQ8800, São Paulo, Brazil) to perform a counterclockwise movement to remove the implants, increasing the torque until the rotation of the implant inside the bone tissue completed the disruption of the bone-implant interface. The maximum torque required to move the implant was considered the removal torque value (Ncm^2^) (Figure 2).

### Histometric analysis

Fragments of the iliac bone with the implants were submitted to 4% paraformaldehyde fixation for 48 hours and washed with water before subsequent dehydration in alcohol solution with increasing concentrations. The plastic infiltration was performed with mixtures of glycol methacrylate (Technovit 7200 VLC) and ethyl alcohol, following gradual variations, ending with two infiltrations of pure glycol methacrylate. After the plastic infiltration, the specimens were embedded in resin and polymerized. Subsequently, the specimens were sectioned longitudinally along the main axis of the implant by means of a high-precision diamond disk. The blocks were mounted on an acrylic sheet with Tecnovit 4000 resin (Kulzer, Wehrheim, Germany). Using a micro-etching system (Exact-Cutting, System, Apparatebau Gmbh, Hamburg, Germany), the slides were processed to include a section of approximately 50-70 μm in thickness. The samples were stained with Stevenel's Blue for histomorphometric analysis^13^. This analysis was used to evaluate the amount of bone mineralization in direct contact with the implant surface (%BIC) as well as the fraction of bone tissue area between implant threads (% BBT - bone between threads). The histological images were captured using a DIASTAR (Leica Reichert & Jung products, Germany) optical microscope, set at 2.5- and 10-fold magnification. The images were sent to a microcomputer (Leica Reichert & Jung products, Germany). The analysis was performed by a blinded, calibrated, and trained examiner using image analyzer software (ImageJ, Jandel Scientific, San Rafael, CA, USA).

### Statistical analysis

The sample size was calculated using paired t-tests based on the %BIC data from the study of Faeda et al., 2009 ^(^ ^4^, which evaluated the effect of different implant surfaces on osseointegration in rabbits. The difference among %BIC averages between different implant surfaces to provide a statistically significant difference was 25.9%, with a standard deviation of 8.3 ^4^. Therefore, the use of 8 rabbits per group in each period was sufficient to obtain a study β-power greater than 0.9 and an α of 0.05.

Normal distribution was confirmed by the Shapiro-Wilk normality test. Thus, the paired t-test was used for inferential analysis of the data to compare the different groups in each experimental period. Repeated Measurements ANOVA was applied to compare the different evaluation periods within each group. GraphPad Prism 8 software (San Diego, CA, USA) was used to perform statistical tests, all of which were applied at the 5% level of significance.

## Results

### Biomechanical analysis

There were no differences in implant insertion torques with the different microstructures (Control vs. hydrophilic). A progressive increase in implant removal torques was observed in all groups. Implants with hydrophilic surfaces presented higher values of removal torque than implants with control surfaces at the period of 8 weeks (p<0.05) (Table 1).

**Table 1:** Mean and standard deviation data of insertion and removal torque values in both groups.

	Insertion torque	Removal Torque		
Implant Type/ Period	Initial	2 weeks	4 weeks	8 weeks
Control	30.69 ± 8.94	27.58 ± 11.93^c^	40.39 ± 25.31^b^	52.77 ± 13.49^a^
Hydrophilic	30.74 ± 9.44	25.23 ± 13.62^c^	45.39 ± 14.86^b^	76.13 ± 16.00^*a^

* p <0.05. Higher removal torque was observed for the hydrophilic group - t-paired test. Different letters represent statistically significant levels between the periods of evaluation within each group (a represent the highest values, b represent the second highest values, and c represents the lowest values). Repeated measurements ANOVA complemented by the Tukey test.

### Histometric analysis

**Figure 3:**
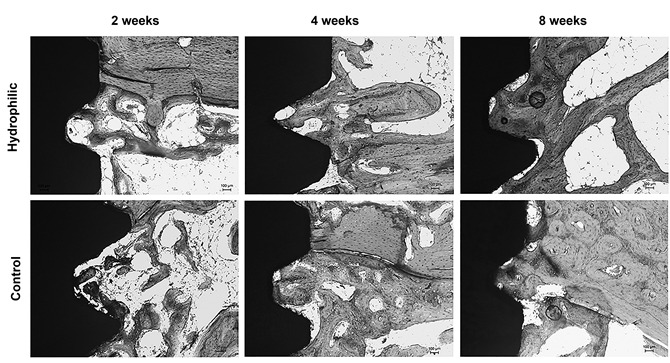
Representative histological images of the non-decalcified sections. It is possible to note a higher degree of osseointegration in the Hydrophilic surfaces at the 2-week period (Stevenel´s Blue Stain. Original magnification 100X).

A progressive increase in the degree of osseointegration was observed on both surfaces with increasing experimental period (p<0.05). Implants with hydrophilic surfaces presented higher %BIC and %BBT values than implants with control surfaces at the experimental period of 2 weeks (p<0.05) (Figure 3, Table 2).

**Table 2:** Mean and standard deviation data of % BIC and % BBT values in both groups.

Analysis	Surface	2 weeks	4 weeks	8 weeks
% BIC	Control	29.49 ± 10.27^b^	41.77 ± 11.91^a,b^	54.80 ± 9.30^a^
	Hydrophilic	42.92 ± 2.85^**b^	53.74 ± 7.54^a^	55.56 ± 4.69^a^
% BBT	Control	23.20 ± 6.75^b^	41.77 ± 6.28^a^	52.36 ± 6.83^a^
	Hydrophilic	34.32 ± 8.52^**b^	47.73 ± 16.16^a,b^	53.22 ± 7.81^a^

** p <0.01. Higher %BIC and %BBT than the hydrophobic group Paired t-test. Different letters represent statistically significant levels between the periods of evaluation within each group (a represent the highest values, and b represents the lowest values). Repeated measurements ANOVA complemented by the Tukey test.

## Discussion

The primary stability of the dental implants directly influences the achievement of secondary stability, however, the findings of this study showed that there was an increase in osseointegration using the hydrophilic surfaces in relation to the control surfaces, despite the equality between the groups in primary stability. Thus, the null hypothesis of this study was rejected.

The primary stability measured through the insertion torque analysis demonstrated that there were no differences in this parameter between the different types of surfaces. In fact, it has been consistently described that these macrostructural features of implants have a more impactful effect on the implant’s stability than the microstructure ^15^ ^,^ ^16^, and the fact that both implants showed similarities in their macrostructure may be the reason for the lack of statistically significant differences in insertion torque between the hydrophilic and control surfaces.

Regarding the conversion of primary into secondary stability, the hydrophilic surface was shown to positively influence this process. According to the findings of this study, the hydrophilic surface increased the %BIC and %BBT values compared to the control surfaces after 2 weeks of implant placement. In addition, implants with a hydrophilic surface showed greater removal torque than implants with a control surface at the 8-week period after implant placement. In fact, hydrophilic surfaces have been shown to accelerate the osseointegration process, due to their wettability property ^14^, as a result of their surface manufacturing process, which is deprived of atmospheric air, reducing the presence of organic compounds in this surface ^17^. This property increases the proliferation of undifferentiated mesenchymal cells ^12^ and their differentiation into osteoblasts ^18^, which stimulates the expression of osteogenic factors on this surface ^11^ ^,^ ^13^. These aforementioned properties demonstrated a positive impact on osseointegration in clinical studies ^9^, and in preclinical studies that evaluated challenging conditions for the osseointegration process, such as in grafted areas ^10^, and in animals with hyperglycaemia ^19^ and osteoporosis ^11^.

An interesting finding of this study is that the differences between the surfaces occurred at different times according to the methods used to assess osseointegration. Histomorphometric analysis makes it possible to more accurately observe the initial stages of the bone tissue formation process, which was evidenced in this study, as the differences were observed at an earlier period of analysis (2 weeks) ^14^. However, the removal torque analysis is influenced not only by the amount of bone, but by its mineralization conditions, where more mineralized bone around the implants increases the mechanical imbrication ^20^ ^,^ ^21^. In fact, although the histomorphometry analysis did not demonstrate differences in later periods, the removal torque analysis demonstrated that hydrophilic surfaces increase secondary stability in the 8-week period, and it is possible that this event is associated with a higher level of bone mineralization around implants with a hydrophilic surface ^11^.

Despite the differences between the surfaces, in general, they both achieved a good osseointegration process, which can be associated with good primary stability of the implants even in low-quality bone. It is also possible that these results were achieved because both investigated surfaces present a pattern that achieves the osseointegration process even better than implants without surface treatment ^10^ ^,^ ^22^. It is important to emphasize that clinically both surfaces have shown good outcomes ^23^ ^,^ ^24^, and that although the surface influences the loading protocol of the implants, after the installation of the prostheses, the surfaces are likely to behave similarly. Then, the positive effect of hydrophilic surfaces can be only clinically relevant in conditions where the immediate loading is not possible.

The current study presents limitations inherent to preclinical studies, such as the challenge of trying to more adequately mimic the presence of low-density bone in the oral cavity and the limited difference in variability between animals. The differences in the environment of the oral cavity and the experimental model used in this study limits the extrapolation of our data in the clinical scenario. It was not possible to check the effect of occlusal forces on the course of osseointegration of the tested implants since the experimental model used in this study impairs the application of a functional load as would occur in the oral cavity in implant-supported prostheses. Furthermore, the method used to assess the secondary stability of implants is not clinically applicable, due to the removal of the dental implants to get the data of this parameter. The resonance frequency analysis could provide information through a more suitable method for clinical application. However, it is important to states that the removal torque analysis have been extensively used in preclinical studies to assess the secondary dental implants stability. Finally, only two implant surfaces were evaluated in the present study, and these findings do not apply to other types of surfaces.

In view of the results obtained, it can be concluded that hydrophilic surfaces accelerate the osseointegration process in low-quality bone even when the implants present good primary stability.
